# Efforts in Bioprospecting Research: A Survey of Novel Anticancer Phytochemicals Reported in the Last Decade

**DOI:** 10.3390/molecules27238307

**Published:** 2022-11-28

**Authors:** Saheed O. Anifowose, Wejdan S. N. Alqahtani, Badr A. Al-Dahmash, Florenz Sasse, Maroua Jalouli, Mourad A. M. Aboul-Soud, Ahmed Y. Badjah-Hadj-Ahmed, Yasser A. Elnakady

**Affiliations:** 1 Department of Zoology, College of Science, King Saud University, Riyadh 11415, Saudi Arabia; 2 Institute for Pharmaceutical Biology, Technical University of Braunschweig, 38124 Braunschweig, Germany; 3 Department of Biology, College of Science, Imam Mohammad Ibn Saud Islamic University (IMSIU), Riyadh 11623, Saudi Arabia; 4 Chair of Medical and Molecular Genetics Research, Department of Clinical Laboratory Sciences, College of Applied Medical Sciences, King Saud University, Riyadh 11433, Saudi Arabia; 5 Department of Chemistry, College of Science, King Saud University, Riyadh 11451, Saudi Arabia

**Keywords:** natural products, anticancer drugs, phytochemicals, chemotherapy

## Abstract

Bioprospecting natural products to find prominent agents for medical application is an area of scientific endeavor that has produced many clinically used bioactive compounds, including anticancer agents. These compounds come from plants, microorganisms, and marine life. They are so-called secondary metabolites that are important for a species to survive in the hostile environment of its respective ecosystem. The kingdom of Plantae has been an important source of traditional medicine in the past and is also enormously used today as an exquisite reservoir for detecting novel bioactive compounds that are potent against hard-to-treat maladies such as cancer. Cancer therapies, especially chemotherapies, are fraught with many factors that are difficult to manage, such as drug resistance, adverse side effects, less selectivity, complexity, etc. Here, we report the results of an exploration of the databases of PubMed, Science Direct, and Google Scholar for bioactive anticancer phytochemicals published between 2010 and 2020. Our report is restricted to new compounds with strong-to-moderate bioactivity potential for which mass spectroscopic structural data are available. Each of the phytochemicals reported in this review was assigned to chemical classes with peculiar anticancer properties. In our survey, we found anticancer phytochemicals that are reported to have selective toxicity against cancer cells, to sensitize MDR cancer cells, and to have multitarget effects in several signaling pathways. Surprisingly, many of these compounds have limited follow-up studies. Detailed investigations into the synthesis of more functional derivatives, chemical genetics, and the clinical relevance of these compounds are required to achieve safer chemotherapy.

## 1. Introduction

The prediction that global cancer incidence by 2040 would rise by 27.5 million new cases compared to the previously recorded 17 million in 2018 (Cancer Research, London, UK) is not overemphasized. Continuous ageing in the population is associated with this rise in the number of new cases. Current measures to deal with this predicament remain largely unsatisfactory due to the setbacks in various treatment methods [1]. Treatments available according to the National Cancer Institute include surgery, radiation therapy, chemotherapy, immunotherapy, hormone therapy, stem cell transplant, targeted therapy, and precision medicine. A recent approach in cancer treatment is photothermal therapy [2]. The choice of treatment depends on the type of cancer developed in the body. The goal of cancer-related research is to detect therapeutics that will have no or minimal side effects and reduce the complexity of chemotherapy.

For the context of the discussion here, lead compounds that have recently been identified from plants are examined. Many drugs available in the market as anticancer agents are secondary metabolites from microbes, marine life, and plants [3]. Natural products such as vinblastine [4], vincristine [5], etoposide [6], teniposide [7], taxol [8], navelbine [9], Taxotere [10], camptothecin [11], topotecan [12], and irinotecan [13] have been approved as chemotherapy agents; all are plant-derived [3,14]. However, the desire for a cancer cure is not really fulfilled, as many of the drugs available have severe side effects [14]. In this regard, it is appropriate to critically review efforts to date in bioprospecting and chemical genetics reports on new plant-derived compounds with increased activity and better therapeutic prospects. We reviewed the efforts in the bioprospecting of natural products from plants against cancers in the last decade.

## 2. Phytochemicals in Bioprospecting Research

The search for bioactive secondary metabolites in plants as anticancer agents is gaining increasing interest from researchers as it offers a more promising future in detecting novel compounds rather than the synthetic approach [15]. Natural products, especially those from plants, have a unique structural diversity, enabling new possibilities in drug research [16]. Traditional medicine has been a reliable source for obtaining useful information about plants with medicinal properties [17]. Some compounds were discovered by accident, i.e., by random screening of plants, microorganisms, and marine organisms or by bioactivity-guided fractionation [18]. Many drug lead structures were discovered through these methods; nevertheless, the precious gifts of nature to humankind still must be used satisfactorily. It is worth noting that among the sources of natural products, plants remain a huge vase of abundant chemical compounds with unprecedented biological activities and mechanistic action. Conversely, only a small portion of the world’s flora has been tested for their potential bioactive compounds [19,20] and surprisingly, drugs derived from plants are particularly low in number, unlike those from other natural product sources [21].

**Figure 1:**
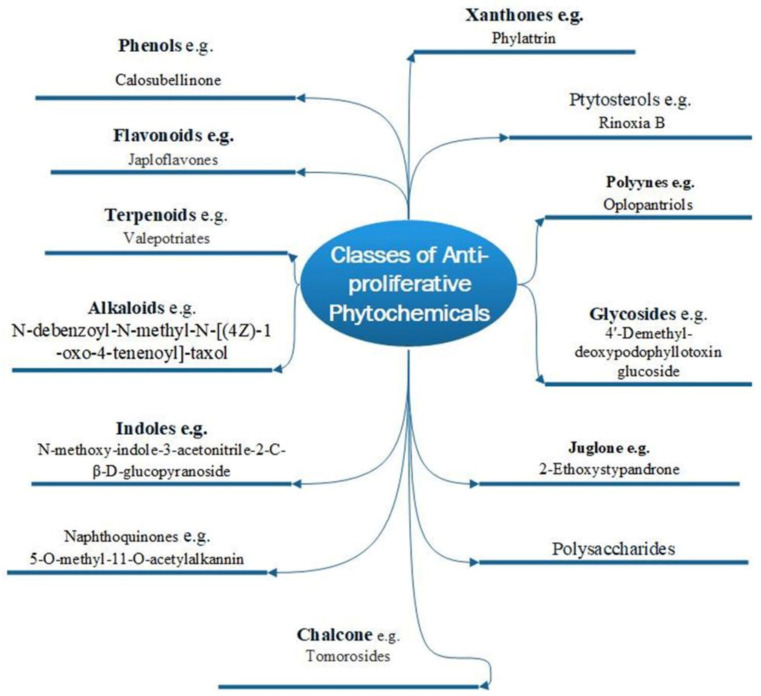
Various phytochemical classes with examples of new members.

There are two basic types of phytochemicals: primary and secondary metabolites. The former class contains the components used for the basic physiological processes in plants, while the latter class is not necessarily used by plants’ physiology but ensures survival in an ecological context. These compounds are known as secondary metabolites, and some have been identified to promote human health and treatment of diseases [22]. Figure 1 shows different classes of plant bioactive secondary metabolites that are of clinical and pharmacological importance, with examples of newly purified compounds that have been studied for their anticancer potentials [23].

## 3. Carcinogenesis and Phytochemicals: Mechanisms of Action and Cellular Targets

**Figure 2:**
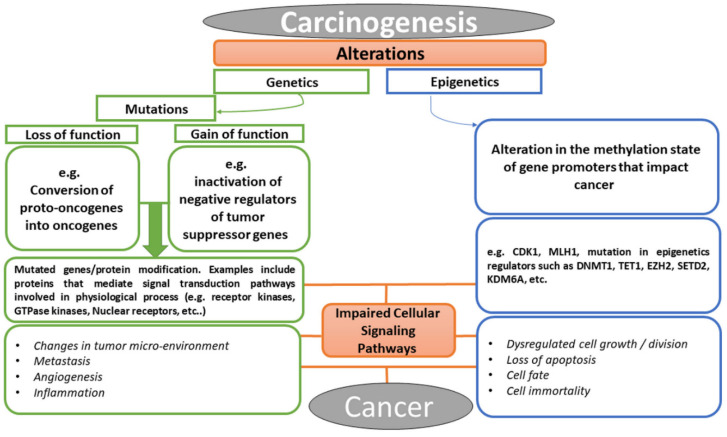
Chronological events that lead to cancer, explaining the relationship between abnormal cellular signaling pathway and cancer formation.

Cancer is a multifactorial disease with diverse etiological factors. The events that lead to cancer arise from the alterations in the genetic constituents of an individual, which may be in the form of mutation, epigenetic alteration, or perhaps the crosstalk between the two processes [24]. As illustrated in Figure 2, the conversion of cellular proto-oncogenes into oncogenes or inactivation of tumor suppressor genes (i.e., loss or gain of function mutations) result in carcinogenesis. Similarly, alteration in methylation or acetylation of promoters of certain genes could consequently result in aberrant proteins or lead to over-expression of some facilitating components of cell signaling pathways. Examples of these facilitators include growth factors and their receptors, such as RTKs, small GTPases, kinases, nuclear receptors, developmental signaling pathway components (e.g., Wnt [25], Hedgehog [26], Notch [27], Hippo [28]), nuclear targets of various signaling pathways (e.g., transcription factors, chromatin remodeler, tumor suppressor genes, cell cycle regulators) [29,30,31,32,33], and lots more [34,35]. These molecular facilitators have been the targets of bioactive compounds used in chemotherapy in the treatment of cancer. Today, the advancement in molecular biology has revolutionized all aspects of research in biology. Efforts in elucidating drug–target interaction have helped in revealing functions of some cellular proteins and in understanding the mechanism of action involved in drug phenotypes [36,37]. Targets of bioactive compounds (e.g., phytochemicals) range from genes to proteins responsible for several cellular processes. Among the notable cellular processes that small molecules target for their anticancer effects are cell death mechanisms (apoptosis and autophagy), metabolic pathways, cell cycle regulation, mitogenic signal transduction, angiogenesis, metastasis, replication, transcription, and translation machinery [38]. Meanwhile, the identification of a therapeutic window for cancer has been a major challenge in chemotherapy in that both the proliferating normal and cancer cells require the same metabolic needs [39]. The selectivity of anticancer drugs is proportional to their ability to have cellular targets that are peculiar to cancer in treatment. Unfortunately, most of the available anticancer drugs in the market lack this pedestal.

Some of the available anticancer drugs are nonselective to cancer cells, targeting housekeeping proteins and genes indiscriminately in both highly proliferating transformed and normal cells [40]. However, most of the metabolic alterations in cancer often result in supporting proliferation (e.g., loss of function mutation in tumor suppressor gene p53). The wide merging in proliferation between transformed and nontransformed cells has been a major drawback to find a therapeutic window in metabolism-based chemotherapy [41].

In recognition of the relentless efforts in bioprospecting plants, reports from many studies have established promising and potent anticancer agents. Many of these phytochemicals are selective in targeting signal transduction pathways that are known to be adulterated in cancer cells [42]. Some of these agents modulate immune systems, control epigenetics and mutations, and leverage the expression of enzymatic products and the blockage of intracellular signal transduction cascades that may lead to the manifestation of cancer. Some other phytochemicals have also been reported to work in synergy with known anticancer drugs, thereby increasing their potency. Some can reverse the multiple drug resistance (MDR) phenotype.

The phytochemicals discussed below are new and have been established in various studies to possess anticancer/antiproliferative properties. These phytochemicals are assigned to their various established phytochemical groups, and the information on whether their mechanism of action and cellular targets is available in the literature was recorded.

## 4. Novel Glycosides

Glycosides include all phytochemicals that have saccharide moieties, i.e., glycosides can occur in any phytochemical class. Prominent among the glycosides with therapeutic values are flavonoid glycosides, anthraquinone glycosides, coumarin glycosides, cardiac glycosides, cyanogenic glucosides, indole glycosides, etc. Efforts in recent times have elucidated novel cellular targets specific to this class of chemicals. Two novel cellular targets, YYI/p65/p300 complex and VRKI/p53BPI, were recently reported to be the target of hyperoside (a flavonol glycoside) and ginsenoside Rg3, respectively [43,44]. The anticancer potency of cardiac glycosides is linked to their inhibition of sodium potassium ATPase (NKA), Ca^2+^ apoptosis induction, sequential activation of autophagy and apoptosis, modulations of topoisomerase II, fibroblast growth factors (FGF-2), and nuclear factor kappa B (NF-ƙB) inhibitions [45]. Nur77 (an orphan nuclear receptor) pathway has also been established as a target of some cardiac glycosides. Nur77 protein is expressed in cardiac glycoside-treated cells, which results in an apoptosis induction. This is due to an alteration in the mitochondria occurring because of the translocation of Nur77 from the nucleus to the cytoplasm [46].

**Figure 3:**
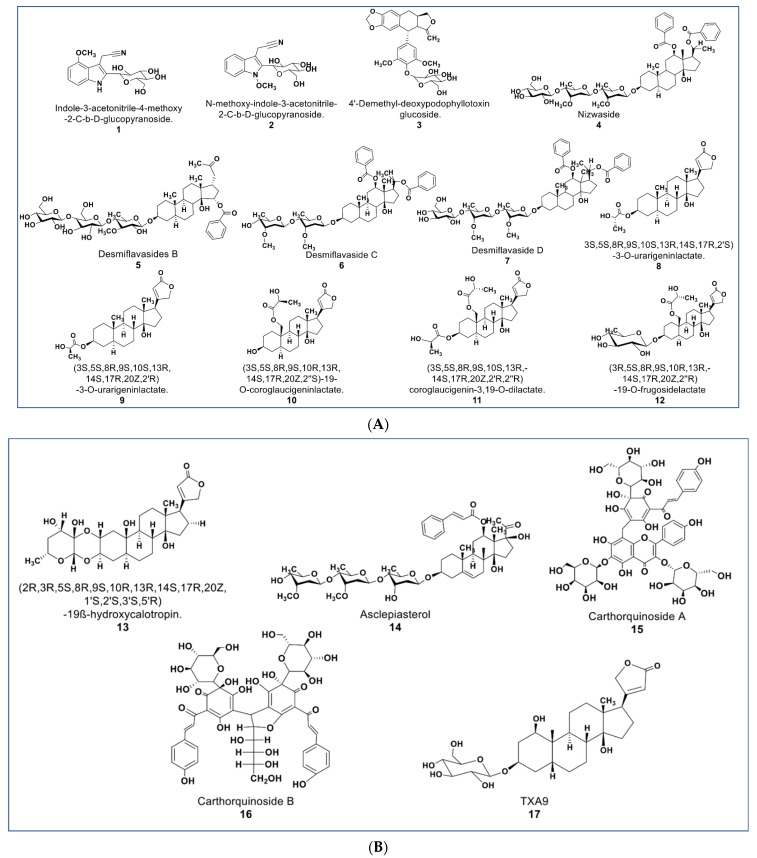
(**A**): Representative members of novel glycosides from various plant sources (compounds **1**–**7** and **12**). Compounds **8**–**11** are cardenolide lactates isolated from *Asclepias curassavica*. (**B**): Representative members of novel glycosides from various plant sources (compounds **13** to **17**).

The chemical structures presented in Figure 3 contain some newly reported glycosides with promising antiproliferative activities, as well as some cardenolide lactates. Some new glucosides (compounds **1** and **2**, Figure 3A) were reported to be cytotoxic against HL-60 and HepG2 (IC_50_ values 1.3 and 2.1 µM; 5.1 and 12.1 µM, respectively). No report was found as to the mechanisms involved in their antiproliferative activities [47]. Another glycoside (compound **3**) reported by Zilla et. al. from *Podophyllum hexandrum* shows cytotoxicity against a panel of cancer cell lines (see Supplementary Table S1) with an IC_50_ value range of 0.208–0.291 µM [48].

As described by Raees et al., some novel pregnane glycosides from *Desmidorchis flava* possess antiproliferative effects against breast and ovarian cancer cell lines [49,50]. Compounds **4**–**7** (Figure 3A) showed cytotoxicity on MDA-MB 231 and SKOV-3 cancer cells [51]. Additionally, compound **4** (nizwaside) showed a stronger antiproliferative effect in MDA-MB-231 cell lines than the known antitumor drug doxorubicin [49]. In contrast, compounds **6** and **7** showed no cytotoxicity on normal breast epithelial cell line MCF-10-2A [51].

A group of potent-to-moderate antiproliferative compounds was isolated from *Asclepias curassavica* [52]. These include four new cardenolide lactates plus one glucoside lactate (compounds **8**–**11**, Figure 3A) and a new double-linked cardenolide glycoside (compound **13**, Figure 3B). While compound **13** showed a strong cytotoxic effect on DU145 cells (IC_50_ 0.29 µM), the cardenolide lactates showed moderate cytotoxicity (IC_50_ value range 1.66–16.96 µM) [52]. Other previously known compounds from this plant were identified to be cytotoxic against DU145 prostate cancer cells. These were six normal cardenolides (IC_50_ values: 0.33–0.92 µM), four double-linked cardenolide glycosides (IC_50_ Values: 0.03–0.28 µM), and some C-21 steroidal glycosides [53,54,55]. Compound **14** (asclepiasterol), a C-21 steroidal glycoside from *A. curassavica*, was reported to modulate the MDR phenotype in MCF-7/ADR and HepG-2/ADM cells at low concentrations (2.5–5.0 µM) and enhancing the cytotoxicity of anticancer drugs [56].

Two new quinochalcone C-glycosides (carthorquinosides A and B, Figure 3B) were isolated from Carthamus tinctorius. Both compounds inhibit inflammation in lipopolysaccharide (LPS)-stimulated HUVEC cells at low micromolar concentrations [57]. A novel cardiac glycoside purified from *Streptocaulon juventas* (compound **17**) showed more potent antitumor activities against NSCLCs (IC_50_ values ranging from 0.006–0.44 µM) than Taxol [58].

## 5. Novel Polyphenolic Compounds

Polyphenolic compounds are generally characterized by the presence of an aromatic ring with hydroxyl groups attached. This phytochemical class is of medical importance to humans as antioxidants, antivirals, and anticarcinogens [59,60]. Polyphenols are subdivided into phenols and flavonoids and can occur as flavonoid glycosides [61]. Several studies have established phenolic compounds as modulators of multiple inflammatory components [62]. Polyphenolic anticancer activity is linked to the modulation of proteins that are involved in procancer signaling pathways, e.g., survival kinases, transcription factors, and growth factors [63]. Reports have demonstrated the role of polyphenols in targeting the PI3K/Akt/mTOR and Wnt/β-catenin signaling pathways and the downregulation of cell cycle regulatory proteins such as cyclins and its kinases. Polyphenols are also known as modulators of epigenetic regulators such as HDAC1 and HDAC2; tumor suppressor proteins p53, PTEN, p21, and NF-kβ, NRF2 and STATs p27 [64,65,66]. Flavonoids share some targets such as MAPK, PI3K/AKT, NF-κB, and NRF2 signaling pathways with phenols [67]. Reports from studies established some flavonoids as antimitotic and microtubule-targeting agents (destabilizers and stabilizers of microtubules) and inhibitors of polo-like kinase 1 (PLK1) [68,69,70,71].

**Figure 4:**
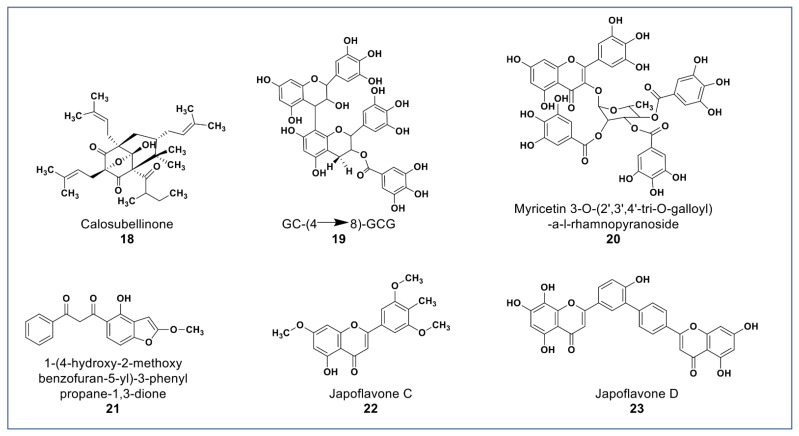
Representative members of new polyphenolic phytochemicals from various plant sources (compounds **18** to **23**).

As shown in Figure 4, novel polyphenols from *Calophyllum soulattri* (compound **18**) showed cytotoxicity against the MDA-MB-231 breast cancer cell line. Compound **18** was almost as active as cisplatin (IC_50_: 19.3 µM) on MDA-MB 231cells. It was found not to be cytotoxic against normal HEK293 cells [72].

A novel proanthocyanidin (compound **19**, Figure 4) purified from *Camellia ptilophylla* proved to be antiangiogenic [73]. This compound suppressed microtubule formation in endothelial cells and cell migration in HMEC-1 cells. It inhibited intersegmental vessels formation (ISV) in Zebrafish and inhibited the phosphorylation of ERK, p38, and Akt in HMEC cells. MAPK and ERK signaling pathways play an important role in angiogenesis [73].

Compounds **20** and **21** (Figure 4) isolated from *Leucaena leucocephala* and *Celosia argentea* were reported to have potent antioxidant scavenging and reducing power, and to be cytotoxic against some cancer cells [74,75]. Compound **20** was reported to have IC_50_ value 2.41 µg/mL against HepG2 [74] while compound **21** (Figure 4A) was reported to have significant antioxidant and cytotoxic activities against Siha, MCF-7, HCT, and HT-29, and no cytotoxicity against Vero cells [74]. In addition to the novel polyphenols, two new flavones (compounds **22** and **23**) isolated from *Lonicera japonica* suggested having hepatoprotective and antihepatoma properties due to activities in SMCC-7721 and HepG2 cells [76].

## 6. Novel Xanthones

**Figure 5:**
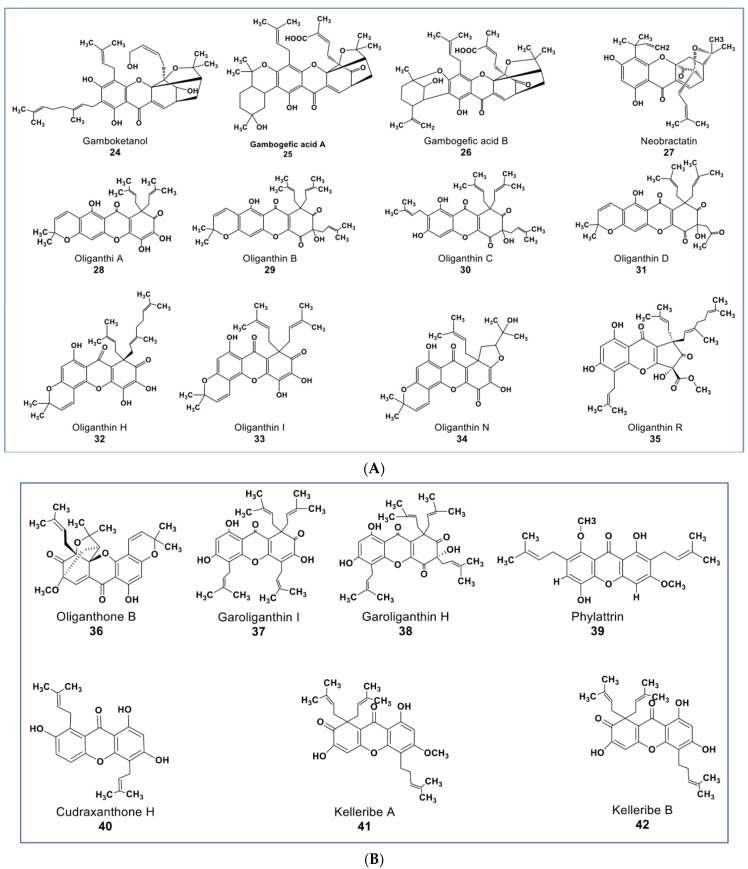
(**A**): Representative members of new xanthone phytochemicals from various plant sources (compounds **24** to **35**). (**B**): Representative members of new xanthone phytochemicals from various plant sources (compounds **36** to **42**).

Xanthones are polyphenols that belong to the flavone subgroup. Many potent anticancer agents have been reported from this group (as shown in Figure 5A,B). The genus Garciniaea produces many anticancer xanthones [77,78,79,80]. Compounds **24**, **25**, and **26** (Figure 5A) are new xanthones that have not received due attention, though they have potent anticancer activities. These compounds were purified from *Garcinia hanburyi* and were reported to show cytotoxicity on cervical carcinoma cells with IC50 values of 3.82, 2.11, and 1.73 µM, respectively [79]. A caged-prenylxanthone (compound **27**) isolated from *Garcinia bracteata* showed cytotoxic effects against a panel of cancer cell lines including A549, MCF7, SMMC-7721, SW480, and HL-60 cells at IC50 values ranging between 2.02–3.25 µM [81]. Compound **27** has been shown to induce growth inhibition via apoptosis and inhibited autophagy flux in A549 and HeLa cells [80]. Compound **27** also inhibited the growth of A549 and HeLa xenograft in mice a little bit higher than the taxol-positive control via the upregulation of muscleblind-like 2 (MBNL2) and CELF 6 RNA-binding proteins [82,83].

Some new xanthones from *Garcinia oligantha* were reported to be cytotoxic against many cancer cell lines [84,85,86,87]. Compounds **28**–**38** (Figure 5A,B) had IC_50_ values ranging from 1.52–<20 µM against A549, HepG2, HT-29, PC, HL-7702, and HeLa cells [86]. Compound **35** (Figure 5A) showed cytotoxic activities against quiescent prostate cancer cells (LNCaP) that are insensitive to Taxol at 20 µM [84]. Xanthones from other plants source include a novel diprenylated xanthone. Compound **39** (Figure 5B) from *Calophyllum soulattri* was reported to have moderately higher antiproliferative effects across nine different cancer cell lines than the positive controls quercetin and kaempferol (IC_50_ values from 9.2–22.10 µM, see Supplementary Table S1) [88]. Compound **40** from *Cudrania tricuspidata* showed antiproliferative effects on oral squamous cell carcinoma by targeting NF-κB and PIN1 signaling pathways [89]. Among five new compounds reported from *Hypericum kelleri*, compounds **41** and **42** (Figure 5B) showed potent cytotoxicity against HeLa cells with an IC_50_ value of 2.5 and 5.9 µM, respectively [90].

## 7. Novel Terpenoids

**Figure 6:**
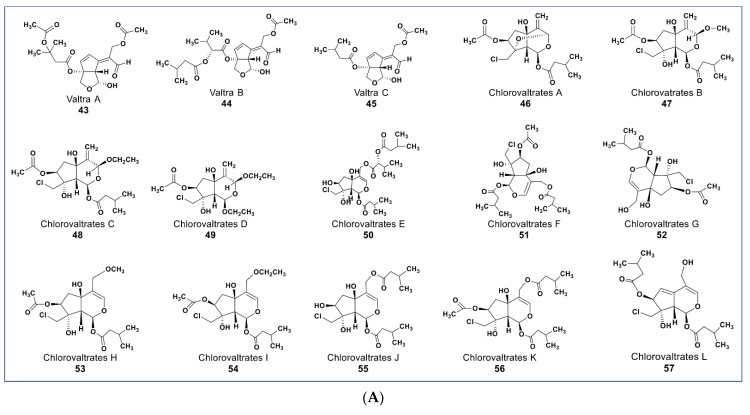

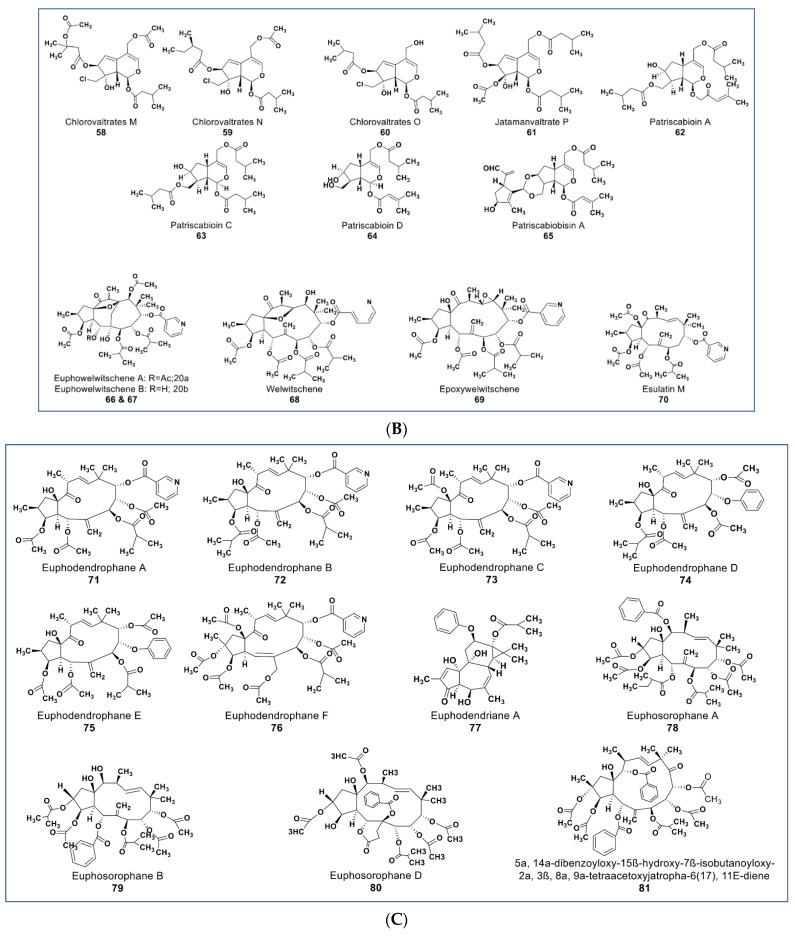

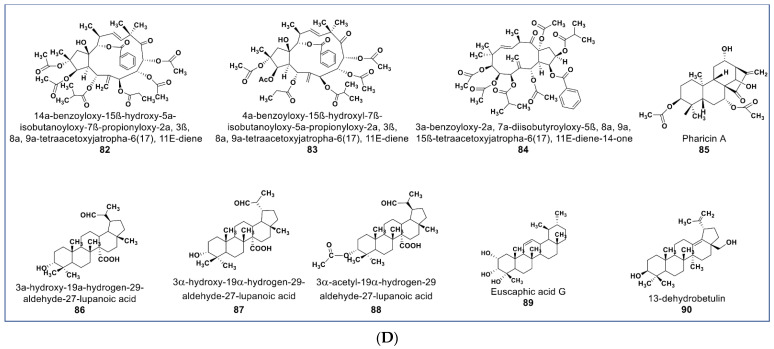
(**A**): Representative members of new terpenoid phytochemicals from various plant sources (compounds **43** to **57**). (**B**): Representative members of new terpenoid phytochemicals from various plant sources (compounds **58** to **70**). (**C**): Representative members of new terpenoid phytochemicals from various plant sources (compounds **71** to **81**). (**D**): Representative members of new terpenoid phytochemicals from various plant sources (compounds **82** to **90**).

Valepotriates, which are triesters of monoterpene alcohol, are known for their excellent anticancer properties [91]. Novel compounds belonging to this chemical class include compounds **43**–**45** (Figure 6A) isolated from *Valeriana jatamansi*. These compounds obtained as degradation products of valepotriates show selective cytotoxicity against PC-3M and HCT-8 cancer cells with IC_50_ values between 2.1 and 6.5 µM [92]. Some new chlorinated valepotriates (compounds **46**–**60**, Figure 6A,B) isolated from *Valeriana jatamansi* were reported to be cytotoxic on several cancer cell lines [93]. Some of these compounds (**56**–**59**) had potent activity on A-549, PC-3, HCT-8, and Bel-7402 cancer cells with IC_50_ values ranging between 1.06 and 10 µM.

Compound **61** (Figure 6B), one of two new iridoids (named Jatamanvaltrate P) isolated from *Valeriana jatamansi*, showed selective anticancer properties in vivo and in vitro [94]. It inhibited the growth of triple-negative breast cancer (TNBC) and MCF-7 cancer cell lines in a concentration-dependent manner (IC_50_ values 4.05–7.05 µM, respectively). Additionally, 61 induced apoptosis and cell cycle arrest in treated cells. It also triggered autophagosome formation, indicated by the upregulation of LC3-II level in treated cells [95]. Some new sets of novel iridoids and bisiridoids were also reported from *Patrinia scabiosaefolia*. Three iridoids (compounds **62**–**64**) and one bisiridoid (compound **65**) showed selective cytotoxicity against HL-60, SMMC-7721, MCF-7, and SW-480, with IC_50_ values ranging from 1.2 to 23.9 µM [96].

Among the major challenges facing cancer chemotherapy is the resistance and cross-resistance of cancer cells to anticancer agents. This menace results from the overexpression of ATP-binding cassette (ABC) transporter proteins such as P-glycoprotein (P-gp) and breast cancer resistance protein (BCRP, ABCG2) “drug efflux transport” [97]. These membrane proteins are responsible for the excretion of drugs from the cell, resulting in low intracellular accumulation and consequent inefficacy of the drug, even at lethal concentration [98,99]. Examples of drugs against which cells have developed cross-resistance are taxanes [100], anthracyclines [101], vinca alkaloids [102], platinum compounds [103], and mitoxantrone [104]. Available MDR modulator drugs such as verapamil [105], cyclosporine [106], dexverapamil [107], tariquidar [108], and zosuquidar [109] show high toxicity and pharmacokinetic interactions with anticancer drugs [110].

Reports from various experiments have shown promising phytochemicals that are more potent than the currently available MDR modulators. Recently, some new jatrophane diterpenoids (compounds **66**–**70**, Figure 6B) were isolated from *Euphorbia welwitschia* [111]. These compounds were reported to be stronger MDR modulators than verapamil [111]. At 2 µM, compounds **68**, **69**, and **70** showed distinct MDR phenotype reversal [112]. Compounds **69** and **70** (Figure 6B) led to selective MDR reversal in EPG85-257RDB-, EPP85-181RNOV-, and EPP85-181RDB-resistant cell lines. Compound **70** showed only minor activity against EPG85-180RDB [111,112].

Some new diterpenoids from *Euphorbia dendroides* (compounds **71**–**77**, Figure 6C) inhibited the growth of a resistant NCI-H460/R lung carcinoma cell line [113] Compounds **71** and **72** both showed strong anti-MDR activities, possessed strong synergy with paxlitatel and doxorubicin, and reversed the resistance to paclitaxel in the NCI-H460/R cell line [114]. Some potent MDR reversal compounds were also isolated from Euphorbia sororia (compounds **78**–**84**, Figure 6C,D) [115,116,117]. Compounds **78**–**80** showed low cytotoxicity at 10 µM against a sensitive MCF-7 cancer cell line but had higher modulation of P-glycoprotein (P-gp) transporter in doxorubicin-resistant MCF-7/ADR cells than verapamil [113].

A novel mitotic arrest inducer belonging to ent-kaurene diterpenoids (compound **85**, Figure 6D) was purified from *Isodon xerophilous*. It was suggested that the compound’s antimitotic activity was via abnormal activation of the mitotic spindle checkpoint protein BubR1 [117]. More importantly, the compound also induced mitotic arrest in paclitaxel-resistant Jurkat and U2OS cancer cell lines [117]. Novel anticancer triterpenes include the three rarely found triterpene derivatives of C-27-carboxylated-lupine (compounds **86**–**88**, Figure 6D) isolated from Potentilla discolor. These compounds demonstrate a higher antiproliferative effect than matrine (a known anticancer agent) against HepG2, MCF7, and T-84 cell lines. In contrast, they seem to be nontoxic against the HL-7702 noncancerous liver cell line at 35 µM [118]. A pentacyclic triterpene (compound **89**, Figure 6D) isolated from *Glechoma longituba* induced apoptosis and cell cycle arrest in NCI-H460 lung carcinoma by targeting the NF-κB/AP-1 signaling pathway [119]. Compound **90** (Figure 6D), a newly isolated betulin derivative from the stem of *Ziziphusspina christi*, showed antiproliferative property against a HepG2 cancer cell line [120].

## 8. Novel Alkaloids

**Figure 7:**
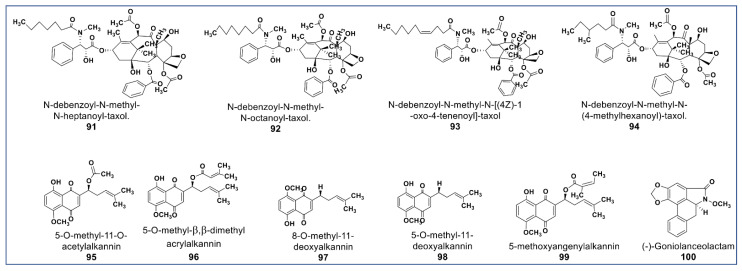
Representative members of new alkaloid phytochemicals from various plant sources (compounds **91** to **100**).

The main phytochemical class, well known for its wide pharmaceutical spectrum, is the alkaloids [121]. Anticancer alkaloids comprise a large number of chemical structures [18]. Prominent among anticancer alkaloids are paclitaxel [122], pyrrolizidines [123], indole [124], quinoline [125], tropane [126], and acronycine alkaloids [127]. Figure 7 shows the chemical structures of some of the newly identified anticancer alkaloids. There are some new taxane derivatives isolated from the ethanol extract of *Taxus wallichiana*. Compounds **91**–**94** (Figure 7) induced a tubulin effect similar to paclitaxel and had antiproliferative activities at IC_50_ values between 0.077 and 7.48 µM against MCF -7, A549, 3-AO, and normal HUVEC cells [128].

Another phytochemical group of pharmacological importance are the naphthoquinones [129]. Some naphthoquinones have been reported as typical endoplasmic reticulum stressors (ERS) [130,131]. Studies have shown that ERS is involved in the activation of inositol-regulating enzyme 1 (IRE1), a representative of unfolded protein regulators (UPRs), which are responsible for proteostasis [132,133]. Persistent ERS induces IRE1 to activate ASK1 (apoptosis signal-regulating kinase 1) and subsequently, activation of the downstream c-Jun N-terminal kinases (JNK) phosphorylation, which induces cell death [134]. Naphthoquinones are potent anti-invasive agents acting on EMT (endothelial mesothelial transition) of cancer stem cells and STAT3 signaling cascades [135,136,137]. Their modulation of ROS enzymes such as ubiquitin-specific protease-2 (USP2) and NADPH Quinone Oxidoreductase 1 (NQO1) gave them selectivity against cancer cells [138]. Compounds **95**–**98** (Figure 7) isolated from *Alkanna cappadocica* showed intriguing cytotoxic activities against a panel of cancer cells including HT-29, MDA-MB 231, PC-3, AU565, HepG2, LNCaP, MCF7, HeLa, SK-BR-3, DU 145, Saos, and Hep3B at low micromolar concentration. Compound **95** showed significantly higher activity in these cell lines than doxorubicin and etoposide. [139]. Moreover, a new phytochemical from Alkanna tinctoria (Compound **99**, Figure 7) was isolated and evaluated for its cytotoxicity against HCT-116 and SW-480 (IC_50_: 4.4 and 9.6 µM, respectively) [123]. A novel naphthoquinone alkaloid (Compound **100**, Figure 7) isolated from *Goniothalamus lanceolatus* showed selective antiproliferative propery that was more potent than 5-fluorouracil when tested against lung and colon carcinoma cells [140].

## 9. Novel Chalcones

**Figure 8:**
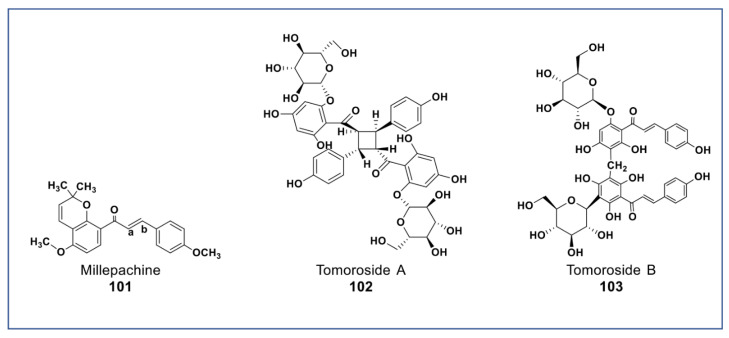
Representative members of new chalcone phytochemicals from various plant sources (compounds **101** to **103**).

A new chalcone isolated from *Millettia pachycarpa* (compound **101**, Figure 8) showed more potent antiproliferative effects (at 2 µM) against HeLa cell than cisplatin [141]. Recently, two newly synthesized derivatives of compound **101** have been reported to have a very potent cytotoxicity than compound **18** in vitro and in vivo (at nanomolar concentration) against many cancer cell lines. These compounds induced apoptosis via G_2_/M arrest, inhibited tububin polymerization, repressed MDR phenotype, and had little cytotoxicity against normal cells [142,143]. Two new chalcone dimers (compound **102** and **103**, Figure 8) isolated from *Helichrysum zivojinii* inhibited the growth of both sensitive and resistant lung carcinoma NCI-H460, NCI-H460/R, and HaCaT cells [144]. Compound **102** suppressed the topoisomerase IIα and significantly enhanced doxorubicin activity (a typical anticancer drug that suppresses topoisomerase IIα) when combined. Compounds **103** increased the efficacy of tipifarnib (farnesyltransferase inhibitor used in the treatment of leukemia) against MDR cancer cells [144].

**Figure 9:**
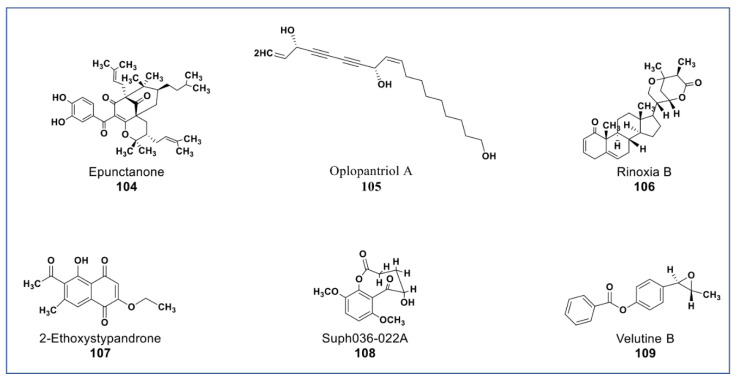
New phytochemicals belonging to different chemical classes. Polyenes, phytosterols, and a juglone analogue from various plant sources (compounds **104** to **109**).

A novel antiproliferative phytochemical belonging to polyprenylated benzophenone (Compound **104**, Figure 9) was isolated from *Garcinia epunctata* [145]. The compound showed anti-MDR effects with relative reversal (RR) values of 0.5, 0.73, and 0.86 in HCT116 (p53−/−), CEM/ADR5000, and U87MG.ΔEGFR glioblastoma cell lines, respectively, and a collateral resistance (CR) value of 0.22 in MDA-MB-231-BCRP cancer cell [146]. A novel polyene (compound **105**, Figure 9) isolated from the root bark of Oplopanax horridus [147] was found to inhibit growth of HCT-116, MCF-7, and SW-480 cancer cells at <10 µM [148]. A new phytosterol (compound **106**, Figure 9) belonging to the withanolides was recently isolated from *Datura inoxia.* This phytosterol was reported to inhibit the growth of HCT15 lung carcinoma at 4 µM [149]. A juglone analogue (compound **107**, Figure 9) isolated from the root of *Polygonum cuspidatum* inhibited the growth of hepatocellular carcinoma (HCC) and HCC cancer stem cells via the blockage of the STAT3 signaling pathway [150]. New phytochemicals isolated from *Brugmansia suaveolens* (compounds **108** and **109**, Figure 9) showed immunomodulatory potentials against PBMC-immunostimulated MCF7, A549, and HL-60 cell lines via enhanced IL-2 and IFN-γ secretions and IL-1β reduction [151,152].

Finally, polysaccharides from various plants have been reported to have anticancer effects due to their scavenging, antioxidant, and reducing ability, as well as their cytotoxicity [153,154]. A newly isolated polysaccharide from fractions of *Meliato osendan* water extract, named pMTPS-3, inhibited the growth of BGC-823 gastric cancer cells at 400 µg/mL [155]. Another novel polysaccharide, named APP3a, was isolated from *Auricularia polytrichais.* APP3a was reported to be a strong scavenger of free radicals, including hydroxyl, superoxide, and DPPH radicals, along with its strong reducing power [156].

## 10. Conclusions

In summary, phytochemicals in chemotherapy have always had a promising future. Many of the phytochemicals reported have, in one way or the other, laid the foundation for resolving the chemotherapy-related setbacks earlier mentioned. Some have shown selective and higher potency than some of the existing anticancer drugs; some work in synergy with existing anticancer drugs, increasing their effectiveness; and some reverse multiple-drug-resistance phenotypes (a worrying situation in chemotherapy). However, it must be said that new compounds are often declared as potential anticancer agents, although only tests with established cell lines are available. This initially only identifies the compounds as cytotoxic. There are usually no attempts to determine whether they really have the potential to become a cancer drug in vivo. More extensive investigations would have to be carried out much earlier, e.g., with xenografts in mice. However, since the effort is great, more advanced models would also have to be established in vitro to identify promising compounds at an early stage. The available information about the biological activities, the cellular targets, and the names of the sensitive and nonsensitive cell lines to the 109 compounds included in this review are summarized in Supplementary Table S1.

## Data Availability

Not applicable.

## Supplementary Materials

The following supporting information can be downloaded at: https://www.mdpi.com/article/10.3390/molecules27238307/s1, Supplementary Table S1: Chemical classes and biological activities of the phytochemicals.
