# Supplementary material for: Efforts in Bioprospecting Research: A Survey of Novel Anticancer Phytochemicals Reported in the Last Decade
Source: Molecules. 2022 Nov 28;27(23):8307. doi: 10.3390/molecules27238307 (PMC9738008; doi:10.3390/molecules27238307)
Supplement: Supplementary file 1 [file molecules-27-08307-s001.zip › molecules-1949723-supplementary.pdf]

Table S1: Chemical classes and biological activities of the phytochemicals

S/N	Compound Names	Chemical class	Anti-proliferative activities/selective cytotoxicity	Cellular targets
1 - 2	Indole-3-acetonitrile-4-methoxy-2-C-b-D-glucopyranoside.<br>N-methoxy-indole-3-acetonitrile-2-C-b-D-glucopyranoside.	Novel Glycosides and	HL-60 myeloid leukemia, HepG2, Mata (human myeloid leukemia)<br>**No report on the effect on normal cells**	None reported
3	4'-Demethyl-deoxypodophyllotoxin glucoside		MCF7 breast adenocarcinoma, PC-3 (Prostate cancer cells), HeLa (cervical adenocarcinoma) HCT-116 (colorectal carcinoma), HEK-293 (human embryonic kidney)<br>**No report on the effect on normal cells**	Mitotic inhibition and G2/M arrest via Chk 2 mammalian checkpoint kinase inhibition in MCF cells.
4	Nizwaside		MDA-MB 231, SKOV-3<br>**Showed no cytotoxic effect on MCF-10 (Human Breast Epithelium)**	No report found
5- 7	Desmiflavasides B, C and D		MDA-MB 231, SKOV-3<br>**Desmiflavaside C Showed no cytotoxic effect on MCF-10**	No report found
8-11<br><br>12	Novel Cardenolide lactates	Cardenolide lactates<br><br>Glycosides	DU145 prostate cancer cells<br>**No report on the effect on normal cells**	Na^+ and K^+ ATPase inhibitions
13	Double linked cardenolide glycoside		DU145 prostate cancer cells.<br>**No report on cytotoxicity effect in normal cells**	None reported
14	Asclepiasterol	C21 Steroidal Glycoside	MCF-7/ADR and HepG-2/ADM cells<br>**No report on cytotoxicity effect in normal cells**	Inhibit the expression of P-glycoprotein in MDR
15 - 16	Carthorquinoside A<br>Carthorquinoside B	Quinochalcone C-glycosides	HeLa, HepG-2, A549, K562 (human chronic myelogenous leukemia), HCT-116 cell lines.<br>**No report on cytotoxicity effect in normal cells**	Inhibitory activity against topoisomerase I
17	TXA9	Cardiac Glycoside	A549, NCI-H1299 (Human non-small cell lung adenocarcinoma),<br>Lu99 lung giant carcinoma cell, Ltep-α2 (Human lung adenocarcinoma)<br>**No cytotoxicity on HE-lung embryonic fibroblast cell.**	No adequate report on cellular target of the compound.

18	Calosubellinone	Phloroglucinol	HeLa cells<br>**No cytotoxicity against HEK293 (human embryonic kidney cells).**	None Found
19	GC-(4®8)-GCG	Proanthocyanidin	HMEC-1 cells (human microvascular endothelial cell)<br>**No report on cytotoxicity effect in normal cells**	Attenuated the phosphorylation of ERK, AKT and P38
20	Myricetin 3-O-(2',3',4'-tri-O-galloyl)-a-l-rhamnopyranoside	Polyphenol	HepG2<br>**No report on cytotoxicity on normal cells**	None Found
21	1-(4-hydroxy-2-methoxy benzofuran-5-yl)-3-phenyl propane-1,3-dione		Siha, MCF-7, HCT, HT-29<br>**No cytotoxicity effect against Vero cell**	None Found
22 - 23	Japoflavones C and D	Flavonoids	SMCC-7721 and HepG2 cells<br>**No report on cytotoxicity effect in normal cells**	None found
24 -26	Gamboketanol, Gambogefic acid A, B	Xanthones	Cytotoxic effect against HeLa cells<br>**No report on cytotoxicity effect in normal cells**	None found
27	Neobractatin		A549, MCF7, SMMC-7721, SW480, and HL-60 cells<br>**No report on cytotoxicity effect in normal cells**	Targets MBNL2 and CELF 6 RNA binding proteins.
28-38	Oliganthins		A549, HepG2, HT-29, PC and HL-7702, HeLa, LNCaP cancer cells.<br>**No report on cytotoxicity effect in normal cells**	None found
39	Phylattrin		SNU-1, HeLa, Hep G2 (liver), NCI-H23, K562, Raji, LS174T, IMR-32, and SK-MEL-28 cells.<br>**No report on cytotoxicity effect in normal cells**	None Found
40	Cudraxanthone H		OSCC (Oral squamous cell carcinoma cells)<br>**No report on cytotoxicity effect in normal cells**	Inhibition of NF-κκB and PIN1.
41 - 42	Kelleribe A and B		HeLa cells<br>**No report on cytotoxicity effect in normal cells**	None Found
43 - 45	Valtra A, B and C		A549, Bel7402, PC-3M and HCT-8<br>**No report on cytotoxicity effect in normal cells**	None Found
46-60	Chlorovaltrates		A 549, PC-3M, HCT-8, and Bel 7402 Hepatoma cell lines	None Found

			**No report on cytotoxicity effect in normal cells**	
61	Jatamanvaltrate P		MCF-7, MDA-MB-231, MDA-MB-453 and MDA-MB-468 **Low cytotoxicity to human breast epithelial cells MCF-10A**	Triggered autophagy formation and increased LC3-II levels
62-65	Patriscabioins A, C, D Patriscabiobisin A		HL-60, SMMC-7721, MCF-7, and SW-480 **No cytotoxicity towards human normal epithelium cells BEAS-2B**	None Found
66-70	Euphowelwitschine A and B, Welwitschene, Epoxywelwitschene Esulatin M		EPG85-257RDB, EPP85-181RNOV and EPP85-181RDB resistant cell lines **No report on cytotoxicity effect in normal cells**	Selective MDR reversal via efflux activity of ABCB1
71-77	Euphodendrophane A-F Euphodendriane A		NCI-H460, NCI-H460/R, DLD-1, U-87 MG	MDR activity
78-80	Euphosophorane A, B and D	Terpenoids	MCF-7/ADR **No cytotoxicity towards normal HEK293 cells**	Inhibiton of P-gp expression in MDR cancer cells
81- 84	Jathrophane derivatives		MCF-7 and A549 MDR-reversing activity on KBv200 cells	MDR activity
85	Pharicin A		Jurkat, U2OS and Raji lymphocytic leukemia cells. **No report on cytotoxicity on normal cells**	Mitotic spindle checkpoint protein BubR1
86- 88	C-27 carboxylated-lupines		HepG2, MCF7 **No cytotoxicity on HL-7702 non-cancerous liver cells**	None Found
89	Euscaphic acid G		NCI-H460 cells **No report found on its cytotoxicity on normal cells**	Targets NF-κB / AP-1 signaling pathways
90	13-dehydrobetulin		HepG2 cells **No report found on its cytotoxicity on normal cells**	No report found
91- 94	Novel Taxane derivatives	Alkaloids	MCF -7, A549, 3-A **No cytotoxicity on** normal HUVEC cells	Induced tubulin effect
95-99	Alkannin derivatives	Naphtoquinones	HT-29, MDA-MB-231, PC-3, AU565, Hep G2, LNCaP, MCF7, HeLa, SK-BR-3, DU 145, Saos-2, and Hep3B cells **Showed cytotoxicity against normal VERO and 3T3 cell lines.**	None Found

100	(-)-Goniolanceolactam		HT29, hct 116, sw48, Caco2, A549, Calu-1, NCI-H23, NCI-H1299,<br>**No cytotoxic effect on ARPE19, MCF10A, and MRC5 normal cells**	None Found
101	Milepachine	Chalcone	A549, h358, MCF7, MDA-MB 231, Huh7, HepG2, LS513, LoVo, HT-29, MS751, HeLa, A2780, PC3, DU-145<br>**Slight cytotoxicity against MCF-10A, HFF-1, L-02 normal cells**	G2M arrest via β-tubulin
102-103	Tomorisides A and B		Synergistic effect with doxorubicin and Tipifarnib in NCI-H460 and NCI-H460/R cells	Inhibition of *topoIIα* and *hif-1α* expression
104	Epunctanone		HCT116 (*p53*$^{-/-}$) cells, CCRF-CEM cells and HepG2 cells	Apoptosis induction via MMP, increase in ROS production, and Ferroptosis
105	Oplapantriol		HCT-116, MCF7, SW8<br>**No report found on its cytotoxity against normal cells**	None Found
106	Rinoxia		HCT15 lung carcinoma<br>**No report found on its cytotoxity against normal cells**	None Found
107	2-ethoxystypandrone		HCC, Huh-7, Li-7, SK-HEP-1, HepG3B, HepG2<br>**No report found on its cytotoxicity against normal cells.**	STAT3 signaling inhibitor
108	SUPH036-022A		Cytotoxicity in PBMC treated MCF7 and A549 cell lines	None Found
109	Velutine		Cytotoxic effect on B16F10, HL-60, HCT116, MCF-7 and HepG2),	None Found
